# Annual Report of the Council

**Published:** 1856-10

**Authors:** 


### TRANSACTIONS

OF THE

### EPIDEMIOLOGICAL SOCIETY OF LONDON.

---

### ANNUAL REPORT OF THE COUNCIL.

(*Presented to the Society, April* 7, 1856.)

### MEMBERS OF THE SOCIETY.

DURING the last twelve months nine resident members, eleven non-resident members, and eight corresponding members have been added to the Society; three resident members have withdrawn, and the Society has suffered by death the loss of a Vice-President (Dr. Hough), and of four other members, of whom three (Dr. Gavin, Mr. Pilcher, and Mr. Walsh) were members of Council.

The following is the present number of resident, non-resident, corresponding, and honorary members:—resident members, ninety-seven; non-resident members, thirty-nine; corresponding members, eighty-five; honorary members, six.

### PROGRESS OF THE SOCIETY.

It was intimated to the Society, at the last Annual Meeting, that the Council had resolved to appoint a Sub-Committee to draw up a brief statement of the objects, proceedings, and results of the Society, for the purpose of distribution among the profession, and general public, and thus making the Society more generally known. This Sub-Committee shortly afterwards presented to the Council a plain and clear summary of the objects for which the Society was instituted, the progress it had made, and its claims to public support.

Nearly three thousand copies of this summary have been circulated among the general and professional public; and through the kind liberality of Dr. Richardson, it has had the benefit of extensive publicity in the columns of his *Journal of Public Health.*

A circular letter, enclosing a copy of the summary, is now also being forwarded to every new Medical Officer of Health appointed under Sir B. Hall's Act; and the Council earnestly hope that many of those gentlemen may be induced to join the Society, where the results of their labours and experience can be brought forward and discussed at the ordinary meetings, with mutual benefit to the Society and to themselves.

### PAPERS READ AT ORDINARY MEETINGS.

The Papers read during the year at the Ordinary Meetings have been of more than ordinary interest.

*Monday, April 2nd,* 1855. A Paper "On Typhoid Fever as it appeared in the towns of Chatham and Rochester in 1854", by Frederick James Brown, M.D., of Chatham, was read by Dr. McWilliam. On the same evening, a Paper "On the occurrence of Fever at Sible Hedingham, Essex, and at Cowbridge, Glamorganshire", by W. Camps, M.D., was read by the author.

*Monday, May 7th.* A Paper entitled "Some Remarks on the Bowel Disease prevalent in the Crimean Expedition during the Winter of 1854-5", by William Smart, M.D., Surgeon of H.M.S. *Diamond,* stationed at Balaclava, was read by Dr. McWilliam.

*Monday, June 4th.* A Paper "On the Propagation of Cholera through the Medium of Water", by John Snow, M.D., was read by the author.

*Monday, July 2nd.* "An Account of the Epidemic of Small-Pox in Jamaica in 1832", by Dr. Bowersbank, of Spanish Town, Jamaica; with "Observations on the Protective Value of Vaccination in Hot Climates", by E. C. Seaton, M.D., was read by Dr. Seaton.

*Monday, August 16th.* Communications "On Cholera", by Dr. Varrentrapp, of Frankfort-on-the-Maine; and "On the Premonitory Diarrhœa of Cholera", by George Todd, Esq., of West Auckland, were read by Dr. McWilliam.

*Monday, November 5th.* An Introductory Address "On Animal Effluvia, and their Effects on Health", by R. D. Grainger, Esq., F.R.S., was delivered by the author.

*Monday, December 3rd.* A Paper "On Puerperal Fever", by Professor Murphy, M.D., was read by the author.

*Monday, January 7th,* 1856. A Paper "On the Prophylaxis of Cholera by some of the Vegetable and Mineral Acids", by J. H. Tucker, Esq., was read by the author.

*Monday, February 4th.* A Paper entitled "Observations on the Cholera Epidemic that occurred in 1854 at St. Laurent D'Aigonze", by Monsieur Reymond Fallot, translated from the French by Dr. Camps, was read by the translator.

*Monday, March 3rd.* A Paper "On the Pathology of Cholera", by Philip B. Ayres, M.D., was read by the author.

### LABOURS OF COMMITTEES.

*Cholera Committee.* This Committee have received from the Home Office replies to the queries sent out to the Black Sea fleet: but from some cause, as yet unexplained, the replies from the Baltic fleet have not yet come to hand. The analysis of the papers from the Black Sea has been left by the Committee in the hands of Drs. Babington and McWilliam, whose report will be published in the *Transactions* of the Society.

*The Hospitals Committee* have received a number of replies to the queries issued by them; but from the extensive scope of the inquiry which the queries embrace, it has been considered that the observations of one year, or even of two years, however carefully recorded, cannot form a reliable basis for a report upon so large and so important a subject. The Committee have, therefore, recently issued further sets of their queries to the naval and military hospitals at home and abroad, as well as to the metropolitan and provincial hospitals of the kingdom.

*Epizootic Committee.* The Report of this Committee has been received, and will shortly be published.

*Nurses Committee.* During the last twelve months the Nurses Committee have been employed in obtaining the opinions and assistance of such influential persons as were in a position to further the objects of the Committee. They have corresponded with masters of workhouses, numbers of the clergy, the various visiting societies of the London parishes, etc., and are still in correspondence with some. A deputation from the Committee having had an interview with the Earl of Shaftesbury regarding their scheme, his Lordship consented to introduce them to the Poor-Law Board, in order there to explain the views and objects of the Committee. The Committee have also prepared the formulæ of such certificates, registers, etc., as would be required in the various workhouses, should the scheme they propose be followed out.

*Small-Pox and Vaccination Committee.* Since the last Annual Report of the Council, the Committee on Small-Pox and Vaccination have exerted themselves in pressing upon the Government the adoption of the principles put forth in the "Memorial on a proper State Provision for the Prevention of Small-Pox and Extension of Vaccination", which was presented by the Council to the General Board of Health in February 1855, and in urging on them the necessity of introducing a Bill to carry these principles into effect. Although, for various reasons, the Government were not able to take up the subject during last session of Parliament, they admitted its great importance, and the necessity for their early interference; and almost on the first night of the present session the President of the General Board of Health gave notice of his intention to introduce a Government Vaccination Bill, and this Bill has since been introduced and read a second time. In the preparation of it the suggestions contained in the memorial of the Council, just alluded to, received full attention, and every disposition was manifested on the part of the Board of Health to give them effect. It is certainly a matter of great regret to the Council that in one important, and indeed fundamental particular, their views have not been carried out, and that Public Vaccination is not separated at once and altogether from the Poor-Law administration. But in other respects,—in transferring the superintendence from the Poor-Law Board to the General Board of Health,—in providing for medical supervision,—in extending the compulsory provisions of the present law, and supplying a working machinery,—and in increasing the remuneration of the public vaccinators, and thereby stimulating them to greater exertion, so much is done to secure more extended and more efficient vaccination, that the Bill is well worthy the zealous support of the Society, and of the profession at large. And the Council, feeling that its introduction by the Government is certainly, in great measure, due to the exertions they have made, think that if this Bill should pass into a law, they will have good reason to congratulate themselves on the result. The Council have suggested certain amendments of detail in a report recently presented to the Society, and adopted by it, and means are now being taken to give these suggestions effect.

### COMMUNICATIONS FROM ABROAD,

The Foreign and Colonial Secretaries continue to receive communications of value from their correspondents abroad, and in the colonies.

Some interesting official papers on cholera have been forwarded to the Society by the Government Board of Medical Officers at Christiana, in Norway; and a communication on the same subject has been received from Dr. Leviseur, Government Physician at Posen, in Prussia. Several valuable papers on the progress of cholera and yellow fever in Brazil have, through the courtesy of the Earl of Clarendon, been received by Dr. McWilliam.

It is to be regretted that this last named country after a century's immunity from epidemic disease in any form, was invaded by yellow fever in 1850, and has recently suffered and is still suffering very severely from cholera. With a suddenness of invasion, and capriciousness of progress not unusual, this scourge made its appearance at Para, situated near the equator, on the 25th of May last, and, spreading to the surrounding districts, speedily ascended the Amazon, becoming, however, milder in its progress up the river.

About the middle of July, or seven weeks after the outbreak of the disease at Para, cholera appeared at Bahia, skipping over Maranham, and all the other intervening ports, on a coast extending over between seven and eight hundred miles; and a few days later it had reached Rio de Janeiro, the capital of the empire, in lat. 23° S. At both places its ravages have been severe. During the months of September and October, the mortality in Rio alone averaged from sixty to seventy deaths a-day.

The total mortality in Rio de Janeiro up to December 4th, 1855, was as follows:—In the white population, amounting to 142,403, the deaths from cholera were 1,131; in the coloured population, amounting to 124,063, the deaths from cholera were 2,368. In other words, the whites died in the ratio of 1 in 126 of the population, while the coloured people lost 1 in 52·4; a result the reverse of what took place during the yellow fever visitation; for on that occasion the whites died in a proportion immensely beyond that of the blacks. From later accounts received from Consul Cowper, cholera and yellow fever were committing great ravages at Pernambuco.

Some mortuary returns, and other documents, have been sent to the Society by the Medical Board of Bombay.

The Council expect some answers from the Presidencies of India to the queries of the Cholera Committee, which were forwarded to India by the Hon. the Court of Directors.

The Honourable Court, who supplied the Society in 1854 with several valuable manuscript reports, and other printed official documents relative to the epidemic diseases of India, were pleased to inform the Society, in the letter accompanying these documents, that the several Governments of India had been instructed to keep their attention directed to the subject of epidemics. Sufficient time has not yet elapsed for either the Court or the Society obtaining the results of any investigations on this subject, or returns of those meteorological observations instituted for the purpose of obtaining a deeper insight into the atmospheric causation of widely diffused diseases, and in relation to the morbific agencies and seasons, and the isothermal lines and distribution of moisture within the tropical zones throughout India and her dependencies. The results of such topographical and meteorological researches promise so much conducive to the knowledge of epidemiology, that the Society cannot but express a hope that subjects so important and interesting may not be lost sight of by the Honourable Court. The necessities and circumstances of the war, that gave rise to the employment of the medical and military officers of the East India Company's service in the ranks of the Turkish Contingent, brought forward a new field for medico-military observations on epidemics in connexion with India, from which the Society may expect to receive much useful information.*

The conference of Paris, upon which the attention of the world was, for some weeks, earnestly fixed, having resulted in the adjustment of the Eastern question, and in the consequent establishment of peace in Europe, we may now confidently look forward to the time when some harvest will be gathered in from the rich fields of epidemiological inquiry presented by Turkey and the Crimea during the last two years.

The first outbreak of cholera and fever among the troops at Varna in 1854; the sudden and rapidly destructive visitation of cholera in the Black Sea fleet; the reappearance, after a partial subsidence, of cholera with increased violence in our army in the Crimea, shortly after the battle of the Alma; the ravages committed by cholera, dysentery, and other diseases, while the army was besieging Sebastopol; the comparative exemption from disease of the naval brigade employed in the same duty during the terrible winter of 1854-5; and the re-establishment of the same army to a state of health

* Since this Report was written, a large packet, containing replies to the "Cholera Queries", has been received from India by Dr. James Bird, the Secretary for India, through the Honourable the Court of Directors.

which has no parallel in the annals of war*—are matters of transcendent importance to the statesman, the general and the medical historian, which ought to be traced, as far as possible to their true causes; and the Council trust they will receive, as they unquestionably deserve, the most patient, full, and rigorous investigation.

Dr. Bryson has already furnished us with some account of the cholera in the Black Sea fleet; and, as has been already stated, the replies to the queries sent to that fleet will be, in due time, laid before the Society. Various other papers on the same subject have, from time to time, appeared in the medical journals of the day. Much, however, is yet wanted to form a complete "Medical History of the Crimean Expedition."

From the officers of the naval and military medical staffs; from the medical officers serving in the various contingent forces during the recent war; from the physicians and surgeons that were employed in the civil hospitals in the East; and from the sanitary commissioners, one of whom is a most zealous and active member of this Society; from all these the Council hope that, in due time, most valuable additions will be made in relation to the nature, causes, and, consequently the prevention of those epidemics to which armies and other large congregations of men have, in all times, been peculiarly subjected.

### THANKS OF COUNCIL.

The Council have, in conclusion, the pleasure to acknowledge many valuable additions to the library of the Society, upwards of fifty volumes having been presented during the year. They have again gratefully to record their thanks to that unwearied benefactor of the Society, Sir C. Mansfield Clarke, Bart., for another donation of £10 to the funds of the Society. Their best acknowledgments are also due to the general and professional press for giving publicity to their papers, giving abstracts of the proceedings, and in other respects forwarding the objects and interests of the Society; and to the editors of the *Veterinarian*, of the *Assurance Magazine*, and of the *Pharmaceutical Journal*, for the presentation of their respective journals to the library.

---

* This may be allowed to have been said not unadvisedly, seeing that in one of the Weekly Returns of the Health of our Army in the Crimea, within the last two months, amounting to 53,000 men, not a single case of death was recorded.

